# Infrastructure-Based Vehicle Localization through Camera Calibration for I2V Communication Warning

**DOI:** 10.3390/s23167136

**Published:** 2023-08-12

**Authors:** Daniele Vignarca, Michele Vignati, Stefano Arrigoni, Edoardo Sabbioni

**Affiliations:** Department of Mechanical Engineering, Politecnico di Milano, 20156 Milan, Italy; michele.vignati@polimi.it (M.V.); stefano.arrigoni@polimi.it (S.A.); edoardo.sabbioni@polimi.it (E.S.)

**Keywords:** road user localization, roadside unit, image detection, I2V communication, intelligent transportation system

## Abstract

In recent years, the research on object detection and tracking is becoming important for the development of advanced driving assistance systems (ADASs) and connected autonomous vehicles (CAVs) aiming to improve safety for all road users involved. Intersections, especially in urban scenarios, represent the portion of the road where the most relevant accidents take place; therefore, this work proposes an I2V warning system able to detect and track vehicles occupying the intersection and representing an obstacle for other incoming vehicles. This work presents a localization algorithm based on image detection and tracking by a single camera installed on a roadside unit (RSU). The vehicle position in the global reference frame is obtained thanks to a sequence of linear transformations utilizing intrinsic camera parameters, camera height, and pitch angle to obtain the vehicle’s distance from the camera and, thus, its global latitude and longitude. The study brings an experimental analysis of both the localization accuracy, with an average error of 0.62 m, and detection reliability in terms of false positive (1.9%) and missed detection (3.6%) rates.

## 1. Introduction

Computer vision has recently grown quickly thanks to the evolution of computational units and the possibility to train models on field data [1]. One of the most popular applications, also thanks to artificial intelligence advancements, is vision-based object detection, which is employed in many fields, ranging from security in crowded areas [2] to obstacle avoidance in autonomous driving and intelligent transportation system (ITS) tasks in general [3]. Object detection and tracking are in fact fundamental for autonomous vehicles (AVs), which need to be aware of the environment surrounding them in order to take decisions at the driver model level. Furthermore, the development of infrastructure-based object detection systems represents a powerful tool to share information about the traffic situation with nearby vehicles and other infrastructure devices. Roadside units (RSU) can also be intended as integrated systems able to scan the portion of the road where the device is installed by means of cameras, LiDARs, and RADARs and disseminate processed data to different road users [4].

**Figure 1:**
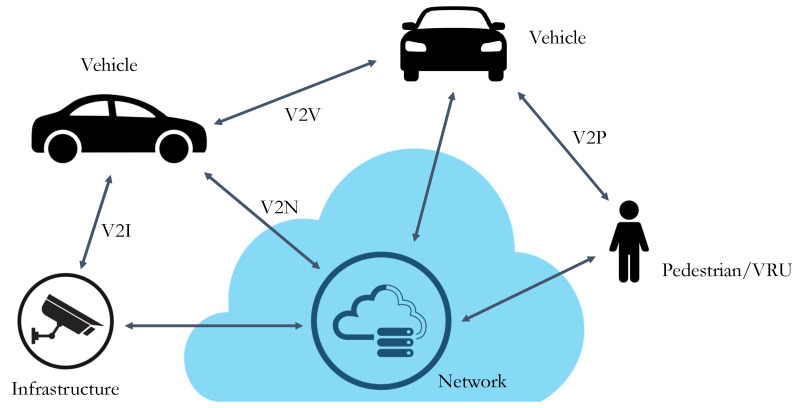
Communication categories for intelligent transportation system.

**Figure 2:**
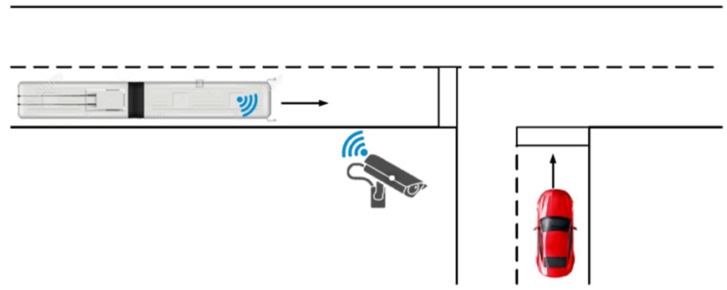
Schematic representation of the scenario under analysis.

The advent of cooperative networks exploiting vehicle-to-everything (V2X) communication (see Figure 1) on the one hand is boosting the improvement of early warning systems, on the other hand, it still presents issues related both to transmission positioning accuracy and communication delays [5]. In this framework, the present work proposes a cheap as well as accurate system for object detection-based localization at urban intersections, depicted in Figure 2, providing real experimental data. This system represents a necessary tool for cooperative ADASs intended to warn, by means of infrastructure-to-vehicle (I2V) communication, other vehicles of potential risks in passing through the intersection.

The main contributions of this paper are as follows:

- The description of a real-world I2V roadside unit configuration for vehicle localization, providing operative performance evaluation and an analysis of delay contributions involved in the entire process.
- A full-scale testing performance analysis of open platforms such as *Gstreamer* and NVIDIA_®_ DeepStream SDK for a camera-based vehicle localization system, assessing both position accuracy and detection reliability.
- A comparison with other state-of-the-art roadside localization methods adopting more complex and expensive systems.

The remainder of the paper is structured as follows: in Section 2, related works about cooperative systems and detection-based localization techniques are reviewed, while in Section 3, the methodology of the proposed system is detailed, focusing on the detection pipeline, the localization algorithm, and the message publication. Section 4 introduces the experimental setup adopted for the testing of the system and the metrics adopted for the comparison of the results with similar state-of-the-art methods. Section 5 reports the results of the system, analyzing detection reliability, localization accuracy, and delays in message generation and communication. Furthermore, a comparison of the proposed system with different methodologies available in the literature is proposed. This provides the possibility to discuss the obtained results in Section 6, highlighting the advantages and the limitations of this research work, and finally, Section 7 draws the conclusions and provides some possible future insights.

## 2. Related Work

Connected and automated vehicles (CAVs) in the future will rely on both onboard sensors and devices installed either on neighboring vehicles or on the infrastructure network, with all these elements being connected, enabling collective perception services [6]. In general, cooperative intelligent transportation systems (C-ITS) aim to exploit connectivity among vehicles and roadside units in order to increase safety and efficiency, especially in urban scenarios.

In [7], cooperative systems related to ITS and infrastructure management are reviewed, being recognized as a crucial element for future smart transportation. In particular, the authors categorize intersections into three clusters, distinguishing between signalized intersections, semi-autonomous intersections, and fully autonomous intersections. In this framework, the focus is pointed on the spatio-temporal reservation and trajectory planning as well as on the intersection goals such as efficiency and safety. As stated in [7], most of the literature available on infrastructure management is related to vehicle-to-vehicle (V2V) communication, while just a smaller portion of the analyzed papers combined V2V and V2I cooperatively. Therefore, a further boost to these topics appears to be necessary.

The review in [8] presents some urban traffic management schemes and an overview of the available traffic sensing technologies, ranging from the inductive loop and microwave RADAR up to video image processing, reporting the advantages and disadvantages for all the analyzed technologies. In particular, for camera-based systems, it is pointed out that it is simple to monitor multiple lanes simultaneously by easily adapting the detection areas. On the other hand, the performance can be affected by occlusion and bad weather and light conditions. The authors in [9] go into detail and investigate the occlusion issue of roadside units involved in V2I systems, discussing the placement scheme for directional sensors such as cameras, and proposing an occlusion degree model to describe the phenomenon.

Cooperative ITS and driving automation have been recently pushed forward, with the development of object detection playing a fundamental role in making them possible, since vehicles need to know what is surrounding them in order to either issue a warning to the driver or actively take action on the vehicle to respond to a dangerous situation; for example, the safety ADAS at an intersection developed in [10]. As far as infrastructure-based object detection is concerned, in [11], four phases are identified for this process: (a) **information collection**, that can be performed with different kinds of sensors; (b) **edge processing** avoiding transmitting a large amount of data on limited bandwidth (e.g., images coming from cameras or point clouds generated by LiDARs); (c) **cloud fusion**, with additional data coming from other connected devices, can be feasible for high-volume low-latency networks; (d) **message distribution** to other road users according to recognized standards. In the literature, it is possible to find a relevant amount of work dealing with object detection systems enabling cooperative perception. As far as data and sensor fusion are concerned, a distinction between late or early fusion schemes for realizing cooperative perception has to be made. Early fusion systems merge raw sensor data before the detection stage, while in late fusion algorithms, each sensor observation is processed independently. The work in [12] proposes both schemes and shows that the early fusion approach guarantees better performances, although there are higher communication requirements in terms of bandwidth.

In the vehicle pose estimation framework, there are many approaches available [13]. They can be classified according to the sensor type, the possibility to fuse different sensors’ data, and the presence of depth information, thus distinguishing between 2D and 3D object detection. Among camera-based systems, ref. [14] presents a 3D object detection and localization based on a k-means-like method which is a clustering algorithm originally developed for signal processing. The work adopts the k-means method on the one hand to classify the contour points of the bottom edge of the bounding box (BB) for better detection, while on the other hand to estimate the pose and the dimensions of the vehicle. Furthermore, pose estimation relies on calculations exploiting intrinsic parameters of camera calibrations, such as focal length and position of the principal point. Then, given the 2D object detection, through the a posteriori probability of vehicle position, orientation, and dimensions the accuracy can be improved.

The authors in [15] propose an automatic traffic camera calibration procedure based on global navigation satellite system (GNSS) to localize the detected vehicle, thus mapping from the 2D world coordinates to the 2D camera coordinate. The approach is built on top of the 2D detection and tracking phase and ground plane coordinates acquisition using GNSS positioning. The core part of the work is the calculation of the transformation matrix obtained through a least squares optimization, solved via a homogeneous linear representation. Another example of vehicle localization based on roadside monocular camera is presented in [16], where a vanishing point calibration [17], together with a system running RANSAC [18] on optical flow vectors obtained from the tracking phase, constitute the vehicle localization algorithm. In addition, to estimate the vehicle states in the 3D world a Kalman filter is proposed based on a six-DOF rigid body model, with covariance matrices empirically determined.

Apart from monocular cameras, roadside cooperative perception systems usually exploit other kinds of sensors such as thermal cameras, RADARs and LiDARs. In [19], the authors propose a late fusion algorithm for data coming from fish-eye and thermal cameras to accurately localize vehicles approaching an urban intersection. The proposed cooperative perception system is split between the roadside unit, in charge of the sensor data acquisition, edge processing, and communication with surrounding vehicles, and the cloud entity, where both images and detection data are stored for continuous model re-labeling and re-training to be then updated on the edge devices. The work in [20] presents a multi-sensor fusion of LiDAR range information and camera semantic information at the data layer in order to issue a blind-spot warning system. After the pre-processing of both images and the point cloud, the latter is projected onto the camera image through a set of rotations and coordinate transformations. Thus, the point cloud is converted from the LiDAR coordinate system to the pixel coordinate system of the camera frame.

As far as cooperative LiDAR-based localization algorithms, Ref. [21] proposes a real-world object perception platform using a single LiDAR installed on a pole in correspondence of an urban intersection. The detection model used is trained on already available datasets collected based on vehicle-equipped LiDAR. Downstream of the detection and tracking phase, objects are geo-localized by the edge computer and data are transmitted to a cloud server which then disseminates the relevant information about the detected vehicle position to the surrounding vehicle and finally the driver through a human–machine interface (HMI). While dealing with LiDAR-based systems, multiple sensors are used in [22], where the authors propose a localization algorithm relying on multiple LiDARs installed at the corner of an urban intersection. The system combines an interacting multiple model (IMM) filter and joint probabilistic data association (JPDA) tracker for vehicle detection and position–velocity estimation.

This paper proposes a simple and reliable solution for 2D object detection and tracking based on a single roadside RGB camera installed at an intersection, as schematized in Figure 2. In particular, the detection and tracking phase is performed through an NVIDIA_®_ DeepStream SDK 5.0 application which adopts a deep neural network based on YOLOv4 Tiny, in a way similar to the one presented in [23], in which the same detection pipeline was used to count people standing at a bus stop. As far as the localization method is concerned, it implements a sequence of linear transformations that uses intrinsic camera parameters obtained from camera calibration to obtain the relative distance of the bounding box from the camera, thus moving to the absolute latitude and longitude of the identified object. Then, the resulting information is included in a standard message for ITS communication (i.e., decentralized environmental notification message, DENM) broadcast to other vehicles which, on the basis of this information, can warn the driver in case of danger. It is worth mentioning that the onboard vehicle control logic for the warning issuing stage goes beyond the expectations of the present paper, thus it is not covered here.

## 3. Methodology

This section is devoted to the description of the system architecture, from the detection and tracking pipeline to the algorithm for localization of the detected objects and the DENM message publication.

**Figure 3:**
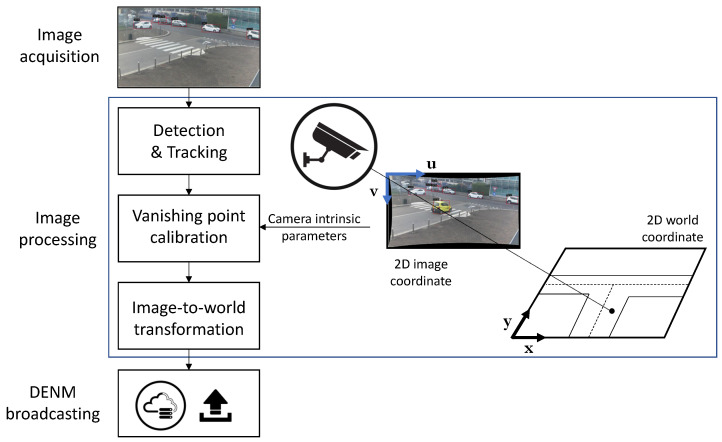
Flow chart of the proposed methodology made of image acquisition, image processing, and DENM broadcasting.

In Figure 3, it is possible to have an overview of the overall framework adopted and presented in the following. After the image acquisition, the processing performed by the computational unit installed on the RSU includes the detection and tracking phase, the vanishing point calibration, and finally the transformation from the 2D image to the 2D world coordinate. The last step performed by the RSU is the DENM message broadcasting via MQTT.

### 3.1. Detection Pipeline

**Figure 4:**
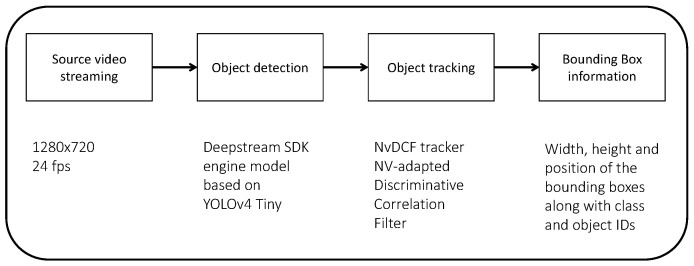
Representation of the *GStreamer* pipeline for detection and tracking phases.

As mentioned, the detection and tracking phases are carried out using an NVIDIA_®_ DeepStream SDK 5.0 application, using a deep neural network based on YOLOv4 Tiny. In particular, these tasks are based on a *GStreamer* pipeline which goes from the image acquisition up to the extraction of the information about the detected bounding boxes as shown in Figure 4. Looking into the details of the items of the pipeline, the starting point is the streaming of the source video (720p) coming from the camera with an acquisition rate fixed at 24 frames per second. Each frame is then passed through the central part of the pipeline, where object detection takes place along with tracking.

In particular, the object tracking is performed using the built-in GStreamer *nvtracker* plugin which tracks detected objects and gives each new object a unique ID. It comes with three possible low-level libraries and for this application, the NV-adapted discriminative correlation filter (NvDCF) is selected. NvDCF tracker uses a correlation-filter-based online discriminative learning algorithm coupled with a Hungarian algorithm for data association in multi-object tracking [24]. Among the pros of this library, on the one hand, it is possible to find less frequent ID switches and high robustness, on the other hand, partial occlusions, shadows, and other transient visual changes may occur.

**Figure 5:**
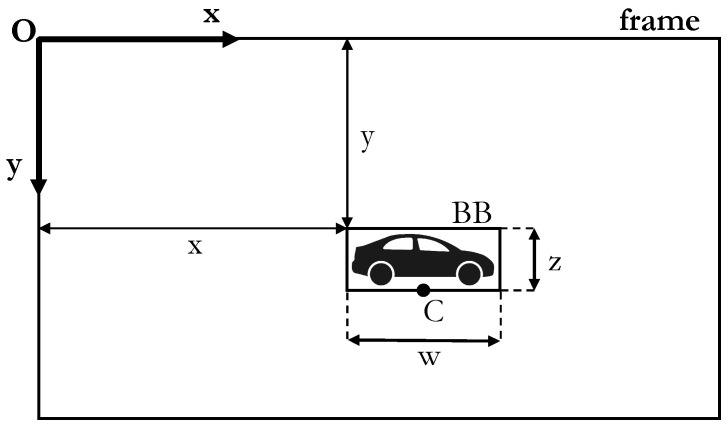
Image reference frame and bounding box representation.

The final part of the detection and tracking pipeline is the reconstruction of bounding box information, which contains (a) the pixel coordinates (*x* and *y*) of the upper left point of the BB in the image reference frame (refer to Figure 5); (b) the dimensions of the BB in terms of width (*w*) and height (*z*); (c) the class of the object (e.g., car, person, bicycle, truck); and (d) the object ID value coming from the tracking.

These data, which are the output of the detection pipeline, represent the inputs for the following localization algorithm that aims to reconstruct the absolute position of the detected object starting from the camera information.

### 3.2. Localization Algorithm

**Figure 6:**
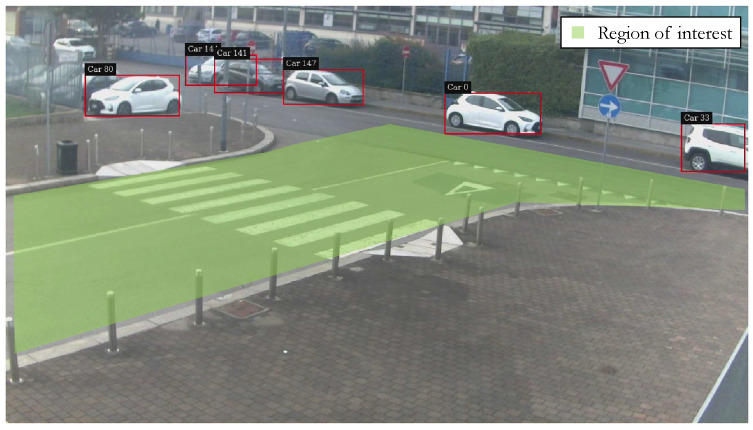
Representation of the region of interest for localization algorithm.

Once the detection data are gathered, the aforementioned BB information is published on the ROS network through a publisher on a dedicated topic. This message is the actual input of the localization algorithm, thus the localization calculations are performed only when a BB is present in a predefined region of interest (ROI) of the intersection considered, as represented in Figure 6.

Moving to the core part of the localization algorithm, the most relevant part is the estimation of the distance of the detected object from the camera using Python OpenCV libraries [25]. The required inputs for the algorithm are:

- intrinsic parameters obtained from the camera calibration, in terms of focal lengths $f_{x}$ and $f_{y}$, and principal point coordinates $c_{x}$ and $c_{y}$;
- camera height $h_{cam}$;
- camera pitch angle ϕ.

Assuming a flat road without any hill and knowing the camera intrinsic parameter calibration values (i.e., $f_{x}$, $f_{y}$, $c_{x}$, and $c_{y}$) as well as the physical parameters of the camera installation (i.e., $h_{cam}$ and ϕ), the solving equation for obtaining the relative distance $x_{loc}$ and $y_{loc}$ of the detected object from the camera is the following:

(1) $$\left[\begin{array}{c} x_{loc}\\ h_{cam}\\ y_{loc} \end{array}\right]={\left[\begin{array}{ccc} 1 & 0 & 0\\ 0 & \cos\phi & -\sin\phi \\ 0 & \sin\phi & \cos\phi \end{array}\right]}^{-1}{\left[\begin{array}{ccc} f_{x} & 0 & c_{x}\\ 0 & f_{y} & c_{y}\\ 0 & 0 & 1 \end{array}\right]}^{-1}\left[\begin{array}{c} x_{pxl}\\ y_{pxl}\\ 1 \end{array}\right]s$$

In Equation (1), $x_{pxl}$ and $y_{pxl}$ represent the non-distorted coordinates of the detected object in the 2D image reference frame, while *s* is a scaling coefficient to be determined while solving the system of equations. It is worth mentioning that the considered point of the detected vehicle (i.e., $x_{pxl}$ and $y_{pxl}$) is set as the mid-point of the bottom edge of the bounding box, represented as *C* in Figure 5, with this point being the closest to the ground and, thus, more realistic for this application.

**Figure 7:**
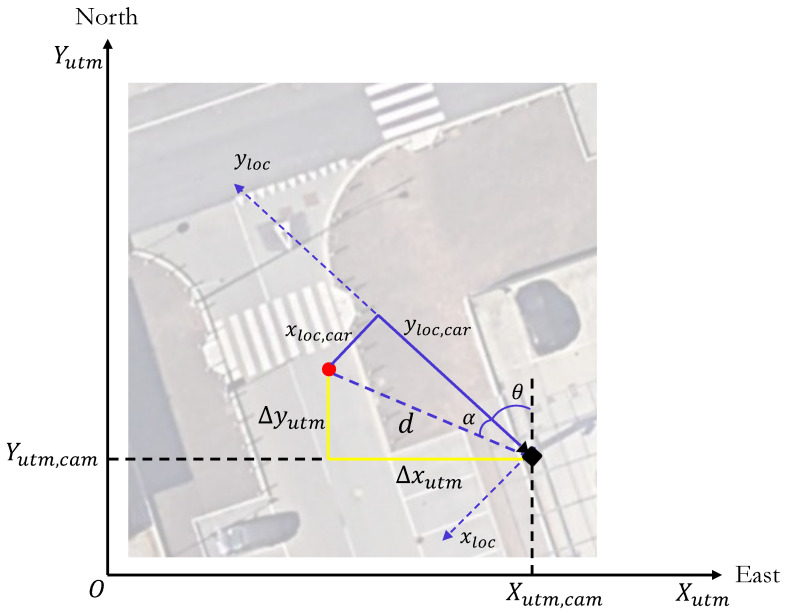
Representation of the UTM coordinate reference frame and the quantities involved in the coordinates transformations.

Once the relative distances of the detected object from the camera are obtained, in order to obtain its absolute position a transformation has to be applied. In particular, a change in coordinates from latitude–longitude–altitude (LLA) to Universal Transverse Mercator (UTM) is required. Figure 7 shows the UTM reference system centered in the camera position, with the x-axis being aligned with the east direction and the y-axis aligned with the north direction. The local reference frame of the camera is also reported, which is rotated by the orientation angle θ, i.e., the absolute angle with respect to the north direction.

Knowing the car’s position in the local reference frame, the distance from the camera *d* is defined as:

(2) $$d=\sqrt{x_{loc}^{2}+y_{loc}^{2}}$$

while the local heading angle α, that is the angle between the $y_{loc}$-axis and the direction identified by the position of the vehicle and the camera itself, can be obtained as follows:

(3) $$\alpha =-\arctan\left(\frac{x_{loc}}{y_{loc}}\right)$$

As a result, knowing all these quantities it is possible to obtain the projections on the UTM axes of the relative position $\Delta x_{utm}$ and $\Delta y_{utm}$ as:

(4) $$\begin{array}{c} \Delta x_{utm}=d\;\sin\left(\alpha +\theta \right) \end{array}$$

(5) $$\begin{array}{c} \Delta y_{utm}=d\;\cos\left(\alpha +\theta \right) \end{array}$$

The last step to obtain the absolute UTM coordinates of the detected car is to add the projection calculated in Equations (4) and (5) to the known UTM coordinates of the camera $X_{utm,cam}$ and $Y_{utm,cam}$, thus obtaining the absolute position of the detected vehicle:

(6) $$\begin{array}{c} X_{utm,car}=X_{utm,cam}+\Delta x_{utm} \end{array}$$

(7) $$\begin{array}{c} Y_{utm,car}=Y_{utm,cam}+\Delta y_{utm} \end{array}$$

Finally, another transformation (i.e., UTM to LLA) is required so that information about the latitude and longitude of the detected object is available for the following DENM message creation.

### 3.3. Message Publication

**Figure 8:**
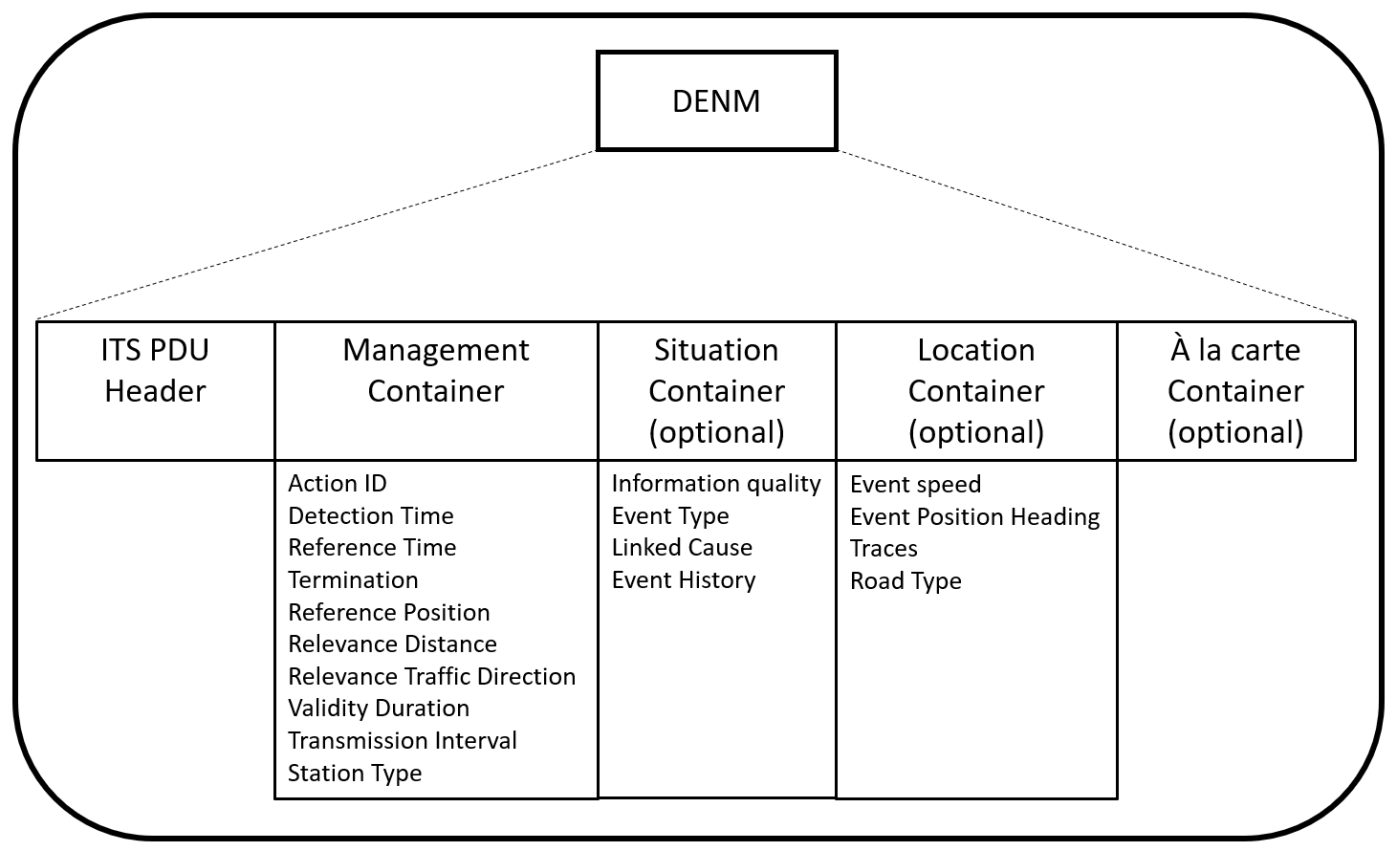
Decentralized environmental notification message structure according to ETSI specification [27].

Cooperative awareness, as already mentioned, can be obtained by gathering information from other vehicles or roadside unit networks. These data have to be conveyed to the recipients with a protocol compliant with a recognized standard in order to support cooperative services which need continuous status information from the surrounding environment. The European Telecommunications Standards Institute (ETSI) proposed a set of messages for ITS and vehicular communication. In particular, two messages are extremely relevant for Day-1 cooperative application systems: the cooperative awareness message (CAM) and the decentralized environmental notification message (DENM). The CAM is specifically intended to be used for V2V communication messages [26] for sharing with surrounding vehicles information about the current position and direction. On the other hand, the DENM has to be used to warn road users about any event, and as a result, it is a much more general kind of message with the payload (schematized in Figure 8) structured as follows [27].

- Management container: includes the timestamp of the event, the source of the message, as well as the absolute position of the event.
- Situation container: includes details about the information quality, the event cause, and history.
- Location container: includes data about speed, heading, path history, and road type.
- “À la carte” container: includes additional specific data for ITS-S application.

According to ETSI, this type of message is designed to be broadcast at a maximum frequency of 10 Hz and they are suitable for intersection collision risk warning (ICRW) use cases according to [28].

DENM message creation and dissemination is the last task for the computational unit installed in the RSU considered in this work for generating warnings about possible dangerous situations at an urban intersection. The transmission protocol selected is message queue telemetry transport (MQTT), adopting for this specific case the public Mosquitto broker [29]. Since the detection and localization algorithm framework is based on ROS, the DENM message is filled in the same framework, having created an ROS custom message shaped according to ETSI documentation. Once the message is created, it is passed through the mentioned *mqtt_bridge* that, given the name of the topics (i.e., both ROS and MQTT) and the type of message, makes available an automatic bidirectional bridge between ROS and MQTT. In this way, the ROS message is converted into a JSON-like message that can be published on the MQTT broker.

## 4. Experimental Setup

**Figure 9:**
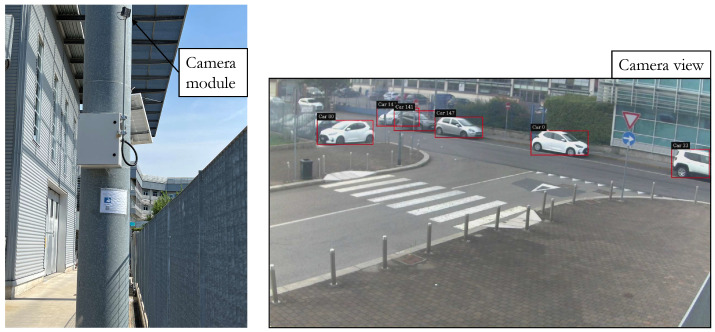
Picture of the roadside unit installed and camera view example.

This section provides a description of the compact and integrated experimental setup which is positioned on a pole at an intersection as shown in Figure 9.

**Figure 10:**
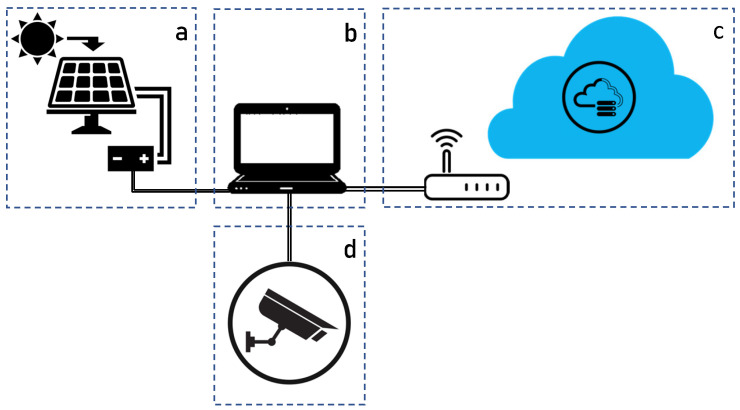
Scheme of the roadside unit components: (**a**) power supply, (**b**) computation unit, (**c**) network, (**d**) camera.

The system schematized in Figure 10 is comprised of four modules: (a) power supply, (b) computation unit, (c) network, (d) camera.

In particular, the power supply module is made of a 12 V automotive battery connected through a solar recharging regulator to a 50 W photovoltaic panel. The computational unit is an NVIDIA_®_ *Jetson Nano* with CPU Quad-core ARM A57@1.43 GHz and GPU 128-core Maxwell, representing a good trade-off between small size and cost, and graphics performance for image detection. Connected to this unit there is a USB3.0 camera module with 48° horizontal FOV, as well as a commercial 4G modem for internet connection.

The architecture of the system is based on a robotic operating system (ROS) as far as image acquisition and data processing are concerned, allowing it to have a simple framework for managing information coming from different sources. On the other hand, once the DENM message is generated, it has to be broadcast to the connected road users. For the experimental tests, a small city car is driven at a known constant speed in a controlled environment situation, with the vehicle being equipped with a global positioning system (GPS) and real time kinematic (RTK) correction to have a ground truth reference localization. Furthermore, in order to have lightweight messaging, a protocol such as message queue telemetry transport (MQTT) [30] is used to send messages in JSON format. As a consequence, the DENM message generated in ROS form has to be converted into a JSON message, so a bridge is used to automatically perform this task [31].

In the following section, the experimental results of the localization algorithm are reported. All the tests were performed during daylight and standard weather conditions. In particular, for the localization testing campaign, a city car was specifically instrumented with an RTK-corrected GPS system in order to have a ground truth value. As far as the detection reliability analysis is concerned, the data were collected by recording a 10-minute long video stream of the regular traffic through the intersection where the RSU was mounted. The most significant frames, i.e., those where a vehicle was actually occupying the intersection, were then extracted for the detection reliability analysis. In addition to the quantitative analysis of the performances of the proposed method, a comparison with a similar sensor configuration method present in the literature is provided. The authors in [15] proposed the normalized root mean squared error (RMSE) as a metric for assessing localization performances. The normalized error is defined as:

(8) $$\epsilon_{i}^{norm}=\frac{d_{i}^{CAM}-d_{i}^{GPS}}{d_{i}^{GPS}}$$

where $d_{i}^{CAM}$ is the calculated distance of the detected object from the camera and $d_{i}^{GPS}$ is the ground truth distance of the vehicle, obtained from the GPS RTK coordinates and the known absolute coordinates of the camera. The RMSE is then calculated as:

(9) $$RMSE=\sqrt{\frac{1}{N}\sum_{i=1}^{N}{\left(\epsilon_{i}^{norm}\right)}^{2}}$$

with *N* being the number of localization points.

## 5. Results

In the Methodology, the algorithmic part for the proposed road user localization by means of an infrastructure-based camera was presented. In this section, the results of the experimental tests are presented. In particular, a focus is dedicated to all three tasks presented in the previous section.

### 5.1. Detection Reliability

As far as the detection phase is concerned, the analysis of the results is focused on the reliability of this task which is fundamental for the subsequent localization part. In fact, since the relative distance evaluation is based on the pixel position of the bounding box, it is important to understand the reliability of the BB information, which represents the output of the detection and tracking phase.

Common issues in image detection are occlusions and poor visibility which lead to missed detection (i.e., so-called false negatives). On the other hand, it happens that in some cases an object is detected even though it is not actually there (i.e., so-called false positives). This is typical in highly dynamic situations in which many objects are close to one another and moving relatively fast.

The analysis is based on the comparison between the number of detected objects in the region of interest, considering just the car class of objects, and the number of objects actually present in the frame. Such a number has been manually obtained by visual checking by the authors for a sample video and checking for the relevant frames in which the region of interest is occupied.

**Figure 11:**
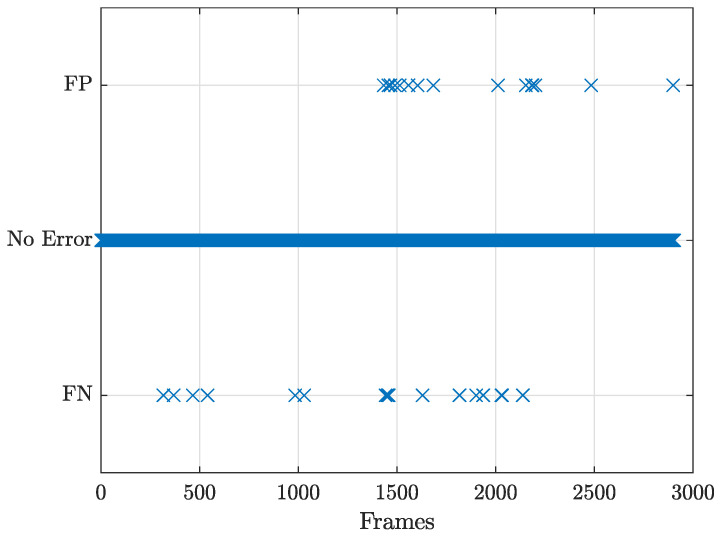
Detection error for cars in the region of interest.

Figure 11 reports the frames in which false negatives (FN) and false positives (FP) occur. The FN condition is faced if the number of visible objects $n_{visible}$ is greater than the number of detected objects $n_{detected}$, thus there has been a missed detection. In the case that $n_{detected}>n_{visible}$, it means that an object is detected even though it is not present, i.e., a false positive case.

Table 1 reports the percentage of false positives and false negatives for car detection, calculated as the ratio between the number of frames with error and the number of frames considered in the sample video. Moreover, the maximum number of consecutive frames in which the detection is not reliable is indicated, as well as the mean value and its variance.

As shown in Table 1, the false negative rate is higher than the false positive one, similar to what is obtained in [19,21], meaning that there is a higher tendency to miss the detection rather than creating false detections. Moreover, when the error is there it may last for a relatively long time. In fact, given the frame rate of 24 fps, in the worst-case scenario, an error lasting for 5 frames is equivalent to about 200 ms duration of missed detection.

As mentioned in Section 2, in the literature there is a relevant amount of studies regarding roadside vehicle localization. In Table 2, a comparison in terms of detection reliability for the proposed method with respect to different and more expensive technologies such as multiple fish-eye and thermal cameras [19] and single LiDAR [21] installed at the intersection is reported.

From the table, it can be seen how the false positive rate is larger than the one obtained in the multiple cameras approach, while it is lower than the LiDAR-based system. On the other hand, the percentage of false negatives is much lower than [21] and still comparable to [19].

### 5.2. Localization Accuracy

This paragraph is dedicated to the analysis of the accuracy of the localization performed with the methodology presented in the previous section. In particular, experimental validation of the model is performed considering as a ground truth a GPS with RTK correction installed on a small city car used for the tests. The tests were performed driving the car in the region of interest at the following constant speeds: 5, 10, and 20 km/h.

**Figure 12:**
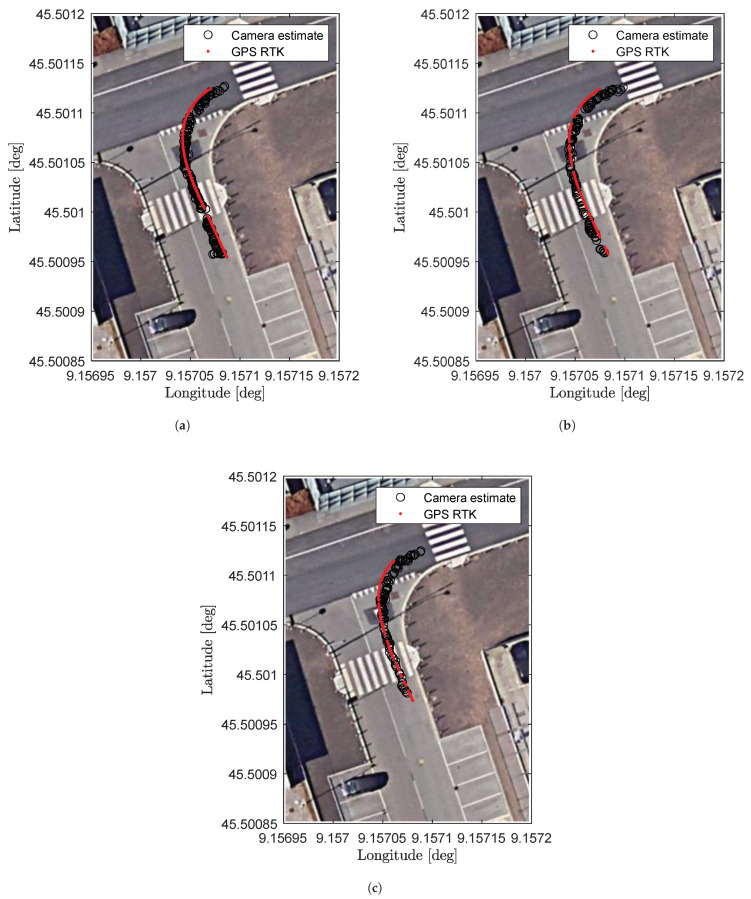
Latitude–longitude positioning comparison between camera estimate and RTK-corrected GPS for different velocities. (**a**) Car speed: 5 km/h. (**b**) Car speed: 10 km/h. (**c**) Car speed: 20 km/h.

In Figure 12, it is possible to compare, for different car speed values, the positioning estimate of the proposed system using the camera (i.e., black circles) and the latitude–longitude coordinates coming from the RTK-corrected GPS (i.e., red points) assumed as ground truth.

As shown in the plots, the localization is quite well reproduced for all the vehicle speeds considered. To perform a more quantitative analysis of the localization accuracy, the estimation error is calculated by moving to UTM coordinates. It is worth mentioning that the GPS data, having a sampling frequency of 10 Hz, have been over-sampled with linear interpolation to match the frame rate of the camera (i.e., 24 fps). Furthermore, in order to have a better understanding of the performance of the methodology presented, the error is split into the longitudinal direction of the vehicle and the lateral one, with respect to the vehicle heading that has been obtained, considering at each time instant two consecutive RTK-corrected GPS points.

**Figure 13:**
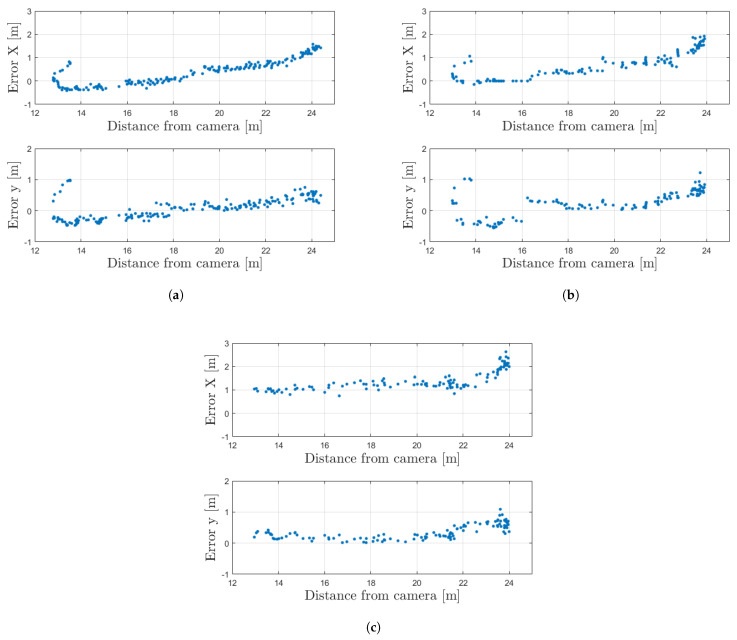
Analysis of the longitudinal and lateral error with respect to the vehicle direction. (**a**) Car speed: 5 km/h. (**b**) Car speed: 10 km/h. (**c**) Car speed: 20 km/h.

Figure 13 shows the point-wise trend of the longitudinal and lateral error with respect to the vehicle for the different speed values as a function of the distance of the vehicle from the camera. As can be seen from the plots, the longitudinal error is positive in most of the cases, meaning that the estimate is ahead with respect to the RTK-corrected GPS position. This is mainly because of the fact that the point of the 2D bounding box chosen (i.e., mid-point of the bottom edge) for the localization estimate does not totally match the GPS antenna position on the car roof. Moreover, it is possible to observe an error increase for all conditions considered when the distance from the camera is greater than 22 m. A similar behavior is visible also for the lateral error. In fact, generally, the farther the object is from the camera, the worse the object detection quality because of a smaller amount of pixels occupied by the object in the frame. As a consequence, the detection performance and, thus, the proposed localization technique that relies on the bounding box points’ positions in the image, is lower.

In Table 3, the root mean squared values of the error trends for the three conditions are reported. In general, a good accuracy of the localization can be observed, with the longitudinal error that seems to have a dependency on the speed of the vehicle, being anyhow bounded below 1.5 m at the highest speed tested. However, thanks to the specific point of the bounding box chosen (i.e., backward with respect to the front of the vehicle), there is always a physical safety factor equal to half of the vehicle length to be considered, thus making the positioning communicated by the infrastructure to surrounding vehicles still conservative. As far as the lateral error is concerned, no significant differences are shown in Table 3, with all values ranging around 0.4 m for all vehicle speeds considered.

Comparing the localization accuracy results of this experimental campaign to some of the works reviewed in Section 2, it is important to note that all of the works adopt the same ground truth source (i.e., RTK-corrected GPS), thus making the comparison reasonable. Table 4 compares the localization performances of the proposed system with both similar and different technologies. The work in [15], similar to the present study, adopts a single RGB camera to reconstruct the vehicle position in the 2D world coordinate system from the 2D camera coordinates by optimizing the transformation matrix based on the acquired data. In [19], a detection system is proposed fusing fish-eye and thermal cameras to localize vehicles at the intersection, while the authors in [21] present a localization method based on a single LiDAR installed on a pole at an urban intersection. A multiple LiDARs method is investigated in [22], showing the capabilities of a system combining IMM and JPDA for vehicle position and velocity estimation.

**Figure 14:**
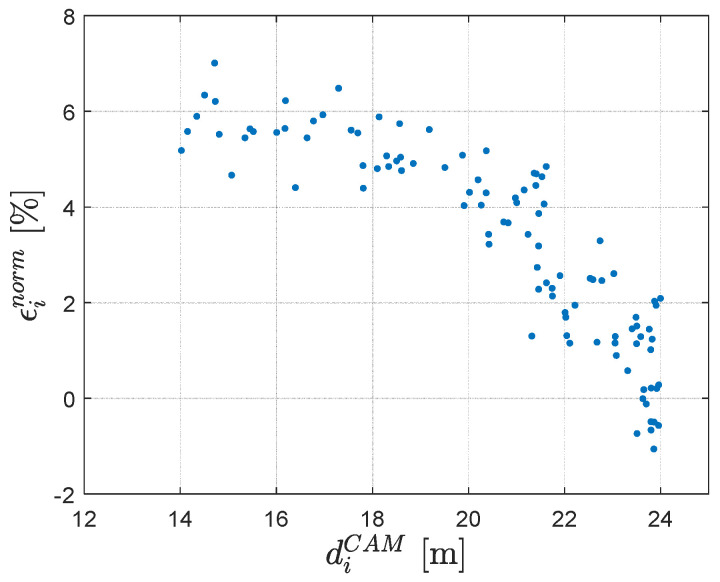
Normalized localization error: car speed 20 km/h.

Furthermore, the work presented in [15] proposes the normalized root mean squared error (RMSE) as a metric for assessing the localization performance. Figure 14 shows the trend of the relative error calculated according to Equation (8) as a function of the distance from the camera $d_{i}^{CAM}$, for the case of a car speed equal to 20 km/h.

Table 5 contains the RMSE, as well as the maximum relative error for the proposed method and the data reported in [15], showing a relevant improvement in this metric.

### 5.3. Delay Analysis

The last part of this section is devoted to an analysis of the total delay, accounting for:

- image acquisition;
- image detection and processing;
- communication delay.

**Figure 15:**
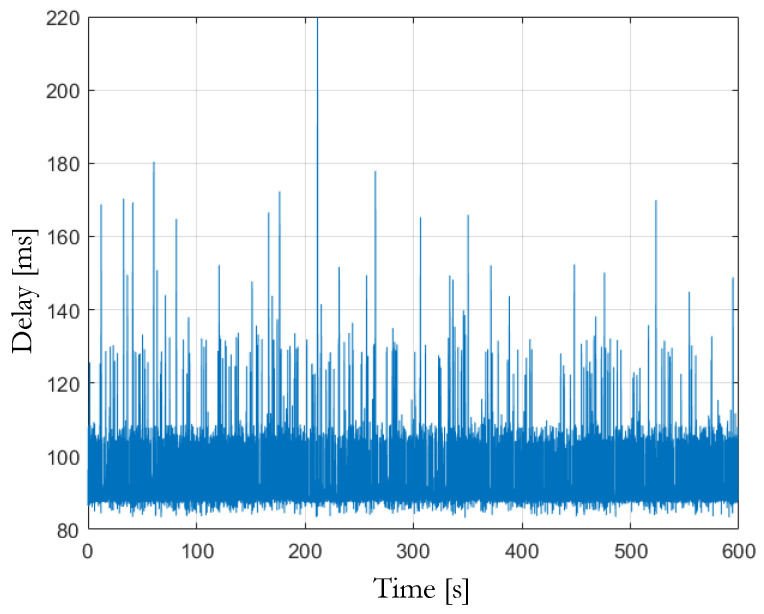
Total delay: composed of image acquisition, image detection, and computational time.

The first two sources of delay can be easily evaluated thanks to the “ROS topic delay” function. Figure 15 shows the trend of the time delay between the image acquisition and the ROS message publication after image detection and processing, with a mean value equal to 98 ms.

To evaluate the image acquisition time, which is a characteristic of the camera module, a glass-to-glass latency test [32] was performed, showing an average time of 55 ms. In this way, it is possible to split the average delay of 98 ms into 55 ms as the average time for image acquisition and the remaining 43 ms as the time required for image detection and processing.

**Figure 16:**
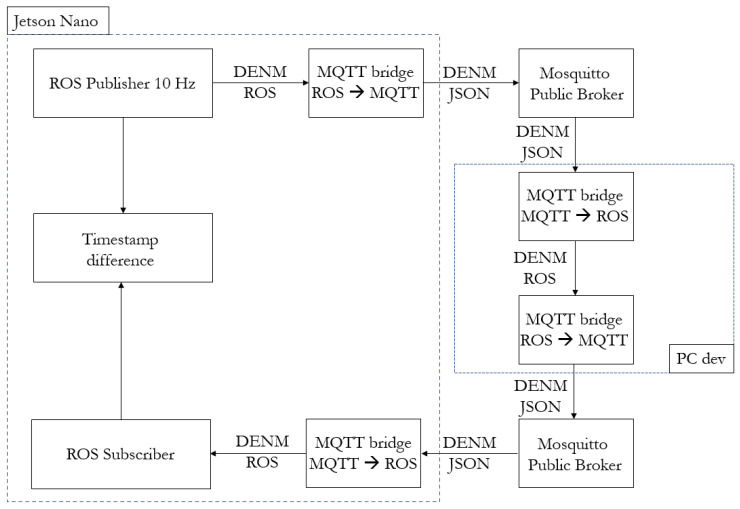
End-to-end infrastructure communication delay loop.

**Figure 17:**
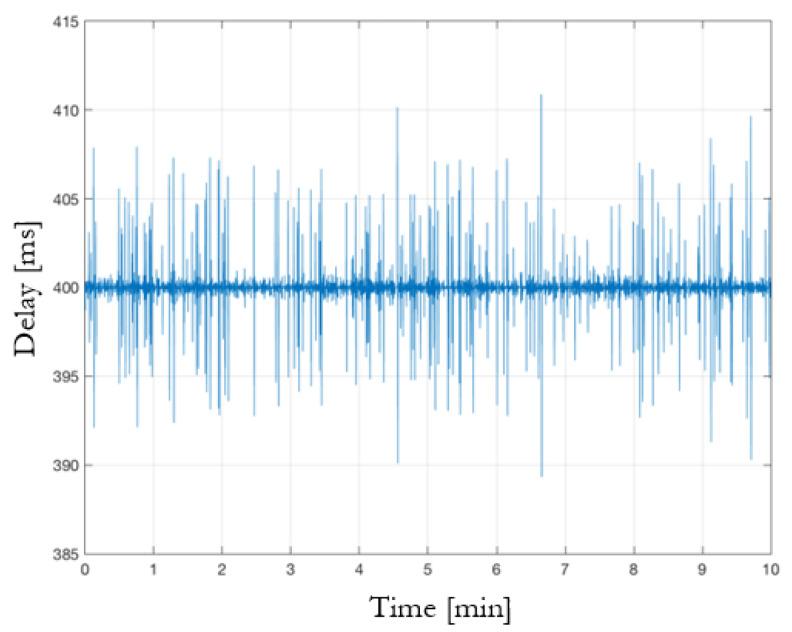
End-to-end infrastructure communication time delay.

As far as the communication delay is concerned, as shown in Figure 16, an end-to-end loop was designed to broadcast a DENM message through the public Mosquitto broker to an external PC, synchronized with the roadside unit. The RSU republishes the same message on a new topic to be visible to the roadside computational unit (i.e., Jetson Nano module), which is then able to calculate the difference between the timestamps of the originating message and the one re-broadcast by the development PC. Therefore, this time difference is equivalent to twice the communication delay. In Figure 17, the trend in the end-to-end communication delay is reported which, by dividing by two, ends up to be on average 198 ms. This quite high value is mostly due to the use of a public online broker and the use of 4G modems for the internet connection, which has been analyzed in detail in [33].

Table 6 summarizes all the sources of delays, with the communication delay proving to be the most relevant one, being responsible for more than half of the total 296 ms delay.

## 6. Discussion

In the previous section, a comparison with works available in the literature [15,19,21,22] proposing different methodologies and technologies for cooperative roadside object localization at the intersection was presented. Thus, the aim of this section is to further discuss the results previously shown, highlighting the positive and the negative aspects of the system proposed in this research activity.

As far as the reliability of the detection system based on an open platform (i.e., *GStreamer* and NVIDIA_®_ DeepStream SDK) is concerned, the performed analysis shows that the detection reliability of the proposed method on the one hand can be improved for both false positive and false negative conditions. On the other hand, the achieved results are definitely good if related to the number of devices and to the cost of the sensors involved in the setup adopted.

When considering the localization performance, the proposed methodology guarantees much higher accuracy than the single camera calibration system presented in [15], while more complex and expensive configurations are able to achieve even better results in terms of average and maximum estimation error. It is worth highlighting that the obtained accuracy is worse than multiple sensor systems using more refined and computationally expensive methods. In fact, the work in [19,21] provides an indication of the latency required for the sensor processing time, perception, and localization algorithms, both around 185 ms, a value much higher than the total delay for image acquisition and detection (i.e., 98 ms) observed and reported in the previous section.

As observed in Figure 13, the quality of the detection and, thus, of the localization worsen as the vehicle moves further from the camera. This is due to the fact that the resolution of the camera is limited to 720p in order to find a trade-off between the detection performances and the costs. In fact, choosing cameras with a higher resolution would require on the one hand the training of a different neural network for the detection algorithm, while on the other hand, the computational burden for the roadside unit would increase, eventually making the overall system not able to run in real-time.

## 7. Conclusions

This paper presents a full-scale experimental testing of a roadside unit (RSU) for vehicle localization based on a USB camera module. The detection pipeline is based on *GStreamer*, making use of an NVIDIA_®_ DeepStream SDK application, while the positioning is obtained with simple transformation procedures based on OpenCV libraries. Experimental validation shows limited false positive and negative rates (i.e., 1.9% and 3.6% respectively) and good localization accuracy, with an error smaller than a similar approach presented in the literature both in terms of absolute (average 0.62 m) and relative error (3.9% RMSE). The comparison with state-of-the-art methodologies indicates how the proposed algorithm has better localization performance with respect to similar studies, while more complex and computationally expensive systems still exhibit a lower estimation error. Furthermore, the analysis of the delays indicates that in the approach proposed in this paper, the most relevant source of latency is the communication layer with the online Mosquitto public broker, while the image acquisition and detection can be limited to less than 100 ms.

These suggest as future developments, on the one hand, to extend the experimental campaign both to assess different roadside unit locations and the performance in pedestrian position estimation, and to provide more statistical data. On the other hand, to explore alternative communication strategies to limit the overall latency, which is crucial for safety-critical applications.

## Figures and Tables

**Table 1:** Detection reliability indexes with error rate, maximum number of consecutive frames, mean number of consecutive frames with error, and variance in the number of consecutive frames with error.

	False Positive CAR	False Negative CAR
Rate	1.9%	3.6%
Maximum consecutive frames	2	5
Mean	1.07	1.87
Variance	0.07	1.45

**Table 2:** Detection reliability comparison with the state-of-the-art methodologies.

	Proposed Method	Multiple Cameras [19]	Single LiDAR [21]
False Positive	1.9%	0.07%	2.59%
False Negative	3.6%	3.45%	16.38%

**Table 3:** Localization root mean squared error analysis.

	Error X (m)	Error Y (m)
5 km/h	0.68	0.35
10 km/h	0.95	0.49
20 km/h	1.47	0.43

**Table 4:** Comparison of localization accuracy with state-of-the-art techniques.

	Proposed Method	Single Camera [15]	Multiple Cameras [19]	Single LiDAR [21]	Multiple LiDARs [22]
Average error	0.62 m	1.6 m	0.27 m	0.14 m	0.1 m
Maximum error	1.05 m	2.5 m	0.50 m	0.32 m	0.5 m

**Table 5:** Relative localization error: RMSE and maximum absolute error.

	Proposed Method	Single Camera [15]
RMSE	3.9%	12.0%
Maximum error	7.0%	18.0%

**Table 6:** Image acquisition, detection, and communication delay analysis.

	Average Process Time (ms)
Image acquisition	55
Image detection	43
Communication	198
Total delay	296

## Data Availability

Not applicable.

## References

[1] Buch N., Velastin S.A., Orwell J. (2011). A review of computer vision techniques for the analysis of urban traffic. IEEE Trans. Intell. Transp. Syst..

[2] Tyagi B., Nigam S., Singh R. (2022). A Review of Deep Learning Techniques for Crowd Behavior Analysis. Arch. Comput. Methods Eng..

[3] Ounoughi C., Yahia S.B. (2023). Data fusion for ITS: A systematic literature review. Inf. Fusion.

[4] Nidamanuri J., Nibhanupudi C., Assfalg R., Venkataraman H. (2022). A Progressive Review: Emerging Technologies for ADAS Driven Solutions. IEEE Trans. Intell. Veh..

[5] Hu L., Ou J., Huang J., Chen Y., Cao D. (2020). A Review of Research on Traffic Conflicts Based on Intelligent Vehicles. IEEE Access.

[6] Masini B.M., Zanella A., Pasolini G., Bazzi A., Zabini F., Andrisano O., Mirabella M., Toppan P. Toward the Integration of ADAS Capabilities in V2X Communications for Cooperative Driving. Proceedings of the 2020 AEIT International Conference of Electrical and Electronic Technologies for Automotive (AEIT AUTOMOTIVE).

[7] Gholamhosseinian A., Seitz J. (2022). A Comprehensive Survey on Cooperative Intersection Management for Heterogeneous Connected Vehicles. IEEE Access.

[8] Nellore K., Hancke G.P. (2016). A survey on urban traffic management system using wireless sensor networks. Sensors.

[9] Du Y., Wang F., Zhao C., Zhu Y., Ji Y. (2022). Quantifying the performance and optimizing the placement of roadside sensors for cooperative vehicle-infrastructure systems. IET Intell. Transp. Syst..

[10] Khayyat M., Arrigoni S., Cheli F. (2022). Development and simulation-based testing of a 5G-Connected intersection AEB system. Veh. Syst. Dyn..

[11] Bai Z., Wu G., Qi X., Liu Y., Oguchi K., Barth M.J. Infrastructure-Based Object Detection and Tracking for Cooperative Driving Automation: A Survey. Proceedings of the 2022 IEEE Intelligent Vehicles Symposium (IV).

[12] Arnold E., Dianati M., Temple R.D., Fallah S. (2022). Cooperative Perception for 3D Object Detection in Driving Scenarios Using Infrastructure Sensors. IEEE Trans. Intell. Transp. Syst..

[13] Arnold E., Al-Jarrah O.Y., Dianati M., Fallah S., Oxtoby D., Mouzakitis A. (2019). A Survey on 3D Object Detection Methods for Autonomous Driving Applications. IEEE Trans. Intell. Transp. Syst..

[14] Guo E., Chen Z., Rahardja S., Yang J. (2021). 3D Detection and Pose Estimation of Vehicle in Cooperative Vehicle Infrastructure System. IEEE Sens. J..

[15] Ojala R., Vepsäläinen J., Pirhonen J., Tammi K. (2022). Infrastructure camera calibration with GNSS for vehicle localisation. IET Intell. Transp. Syst..

[16] Lu D., Jammula V.C., Como S., Wishart J., Chen Y., Yang Y. CAROM-Vehicle Localization and Traffic Scene Reconstruction from Monocular Cameras on Road Infrastructures. Proceedings of the 2021 IEEE International Conference on Robotics and Automation (ICRA).

[17] Kanhere N.K., Birchfield S.T. (2010). A taxonomy and analysis of camera calibration methods for traffic monitoring applications. IEEE Trans. Intell. Transp. Syst..

[18] Fischler M.A., Bolles R.C. (1981). Random Sample Consensus: A Paradigm for Model Fitting with Applications to Image Analysis and Automated Cartography. Commun. ACM.

[19] Zhang R., Zou Z., Shen S., Liu H.X. (2022). Design, Implementation, and Evaluation of a Roadside Cooperative Perception System. Transp. Res. Rec..

[20] Xiang C., Zhang L., Xie X., Zhao L., Ke X., Niu Z., Wang F. (2022). Multi-sensor fusion algorithm in cooperative vehicle-infrastructure system for blind spot warning. Int. J. Distrib. Sens. Netw..

[21] Bai Z., Nayak S.P., Zhao X., Wu G., Barth M.J., Qi X., Liu Y., Sisbot E.A., Oguchi K. (2022). Cyber Mobility Mirror: A Deep Learning-based Real-World Object Perception Platform Using Roadside LiDAR. arXiv.

[22] Srinivasan A., Mahartayasa Y., Jammula V.C., Lu D., Como S., Wishart J., Yang Y., Yu H. (2022). Infrastructure-Based LiDAR Monitoring for Assessing Automated Driving Safety. Proceedings of the WCX SAE World Congress Experience.

[23] Vignarca D., Prakash J., Vignati M., Sabbioni E. Improved Person Counting Performance Using Kalman Filter Based on Image Detection and Tracking. Proceedings of the 2021 AEIT International Conference on Electrical and Electronic Technologies for Automotive (AEIT AUTOMOTIVE).

[24] NVIDIA Gst-Nvtracker. https://docs.nvidia.com/metropolis/deepstream/dev-guide/text/DS_plugin_gst-nvtracker.html.

[25] OpenCV Camera Calibration and 3D Reconstruction. https://docs.opencv.org/4.x/d9/d0c/group__calib3d.html,.

[26] (2014). Intelligent Transport Systems (ITS); Vehicular Communications; Basic Set of Applications; Part 2: Specification of Cooperative Awareness Basic Service.

[27] (2014). Intelligent Transport Systems (ITS); Vehicular Communications; Basic Set of Applications; Part 3: Specifications of Decentralized Environmental Notification Basic Service.

[28] (2018). Intelligent Transport Systems (ITS); V2X Applications; Part 2: Intersection Collision Risk Warning (ICRW) Application Requirements Specification.

[29] Mosquitto E. Eclipse Mosquitto MQTT Server/broker. test.mosquitto.org.

[30] Ahamed J., Zahid M., Omar M., Ahmad K. (2019). AES and MQTT based security system in the internet of things. J. Discret. Math. Sci. Cryptogr..

[31] GROOVE X, I. Mqtt_Bridge. https://github.com/groove-x/mqtt_bridge.

[32] Bachhuber C., Steinbach E. A system for high precision glass-to-glass delay measurements in video communication. Proceedings of the 2016 IEEE International Conference on Image Processing (ICIP).

[33] Prakash J., Vignati M., Sabbioni E., Cheli F. (2022). Vehicle Teleoperation: Human in the Loop Performance Comparison of Smith Predictor with Novel Successive Reference-Pose Tracking Approach. Sensors.

